# Contagion on the Internet

**DOI:** 10.3201/eid0803.010286

**Published:** 2002-03

**Authors:** Trudy M. Wassenaar, Martin J. Blaser

**Affiliations:** * Molecular Microbiology and Genomics Consultants, Zotzenheim, Germany; † Virtual Museum of Bacteria, www.bacteriamuseum.org; ‡ New York University School of Medicine and New York Harbor VA Medical Center, New York, USA

**To the Editor: **Computer viruses are designed to be pests, proliferating in uncontrolled ways and causing severe damage to electronic data. These malignant programs, which amplify between files and computers, are strikingly similar in virulence, modes of spread, and evolutionary pathways over time to the microbes that cause infectious diseases. Both biological viruses and these virtual viruses are transmitted from host to host. Computer viruses are a human invention; however, their development follows a well-recognized biological route. Relatively harmless ancestors gradually or step-by-step evolve into “pathogens;” the host develops adaptive defense mechanisms, which in turn select for new virus “variants;” eventually, equilibrium is reached between infection and host defenses. Comparing “virtual microbes” with their biological counterparts can help us control both. 

 The term “computer virus” is loosely used to describe computer “malware,” an umbrella term that includes the following categories. 1) Viruses. A computer virus is a program that implants a version of itself in any program it can modify. The modified program, once run, attempts to modify other programs directly associated with it. Computer viruses spread by sharing data on infected disks or diskettes. Unlike their biological counterparts (which are fast and very infectious), computer viruses spread slowly and infrequently between computers. 2) Worms. A worm is a self-contained program that replicates itself and sends copies to any connected computer, with little or no user interaction. Unlike biological worms (which spread slowly), computer worms spread rapidly and without much user interaction between computers of a network, including the Internet. (In view of the contagiousness of biological worms and viruses, the terms should have been reversed.) 3) Trojan horses. A Trojan horse is a program concealing harmful code that usually makes a computer or network available to unauthorized users in an appealing or unsuspicious package. A virus, worm, or Trojan horse can be latent (then also called a logic bomb) and become active only after a certain period. 

 Each class of computer malware has hundreds of variants, and many variants have several slightly modified versions, paralleling microbial diversity. Worms, such as the infamous "ILOVEYOU" worm in 2000, may employ a universal message of gratification to entice users. Their wide dissemination parallels the spread of socially transmitted diseases (e.g., influenza) that have the potential to infect everyone susceptible. In contrast, computer viruses (spread by sharing data on infected diskettes) parallel sexually transmitted diseases, whose spread is related to specific behavioral practices. Viruses or worms that are spread undetected but are activated at a later date (as was the case with the Michelangelo virus, discovered in 1991 and still around) resemble latent microbes, such as HIV*.* Denial-of-service attacks, which block access to a server by an onslaught of messages, are the equivalent of toxins, since neither can reproduce in their host and are only harmful above a critical concentration. Spam (unwanted but harmless e-mail), the curse of computer users with slow modems through expensive telephone connections, resembles bacterial commensals that can injure the host only under specific conditions.

 Biological viruses can mutate rapidly, create novel pathogenic and transmission routes, and develop antigenic variation to evade host immunity. In the computer world, worms exhibit similar behavior. Once a worm has been transmitted successfully, variants quickly emerge. These variants cause damage in similar ways but evade detection and impediments installed to provide “immunity” to the original “strain.” Therefore, knowledge of biological infections can be used to predict and anticipate highly virulent computer infections.

 Although the computer user has some recourse against computer viruses, the costs may be high. As with biological viruses, good hygienic practice is helpful. Just as they should wash hands frequently, avoid exposure to people with colds, or use condoms to protect against infectious diseases, computer users should mistrust (and thus not open) files received through unexpected channels or with unknown extensions or subject lines, request confirmation from the sender before opening attachments, and regularly back up hard disks to reduce the risk of losing data. The consequences of such actions in terms of time, disk space, and efficiency illustrate a biological truth: immunity has cost. Effective antiviral barriers are impediments to communication. Moreover, virus protection programs are only as good as the last virus recognized, providing only partial protection at best. Computer users have not always taken inconvenient precautions, even in view of serious consequences. ILOVEYOU was a worm that used the same mechanism of spread as Melissa, which had been released a year earlier. Yet, ILOVEYOU turned out to be even more destructive than Melissa.

 Biological immune disorders in which host defenses turn against the host and actually cause damage are known as autoimmune diseases. Computer autoimmune disorders parallel their biological counterparts. Recently, a warning (defense mechanism used by computer users) turned out to be a not-so-harmless hoax. The hoax warning stated that certain files were infected by a computer virus. Heeding the warning, unsuspecting computer users removed the affected utility files from their computers’ operating systems. The harm mediated by this “host defense” was relatively small in this particular case, resembling the discomfort of allergies, in which immune responses to benign agents cause limited damage. However, more malignant forms of “automutilating” hoaxes are likely to emerge that could be as devastating to computers as some autoimmune diseases are to humans.

 The electronic monoculture that improves communication also increases the risk for contagion. Predominant use of a single operating system has improved communication and sharing of electronic data but has also facilitated ready amplification of virulent programs. As with biological infection, transmission of computer infection depends on susceptibility of the population. Virus producers saw an opportunity in the popular preference worldwide for PCs with Microsoft Windows operating systems. The enormous popularity of these systems, along with their long-recognized inadequate protection against misuse, made computer users susceptible. Virtual viruses able to infect multiple operating systems are rare (as are biological viruses with broad host specificity), and even when infected, computers that run on different operating systems (e.g., Mac, Unix) or other-than-Outlook e-mail programs usually are dead-end hosts for PC viruses.

 Pathogens do not reinvent the wheel. Virulence genes are constantly “stolen” and reused. Thus, new combinations of virulence genes can result in new pathogenic strategies, and such combinations frequently accumulate in pathogenicity islands. Reuse and combination of effective (and infective) strategies are also common in computer malware. A recent example demonstrates the value of just the right amount of virulence. A highly dangerous worm called Nimda (Admin in reverse) was released exactly 1 week after the September 11, 2001, terrorist attack in the United States. Nimda combined the most powerful strategies of Code Red and SirCam and spread more rapidly than any previous worm. Clicking on the subject line of an infected e-mail (to delete it, for instance) itself activated the worm. However, because of the immensity of the threat, the Internet community responded extremely rapidly. Within hours after its release, alerts to system administrators on how to block the worm had effectively slowed its spread. Early surveillance and barrier development averted disaster. As in contained epidemics of hemorrhagic fevers, the immense threat of high contagion and lethality prompts effective measures to rapidly recognize outbreaks and prevent pandemics. 

 The types of measures to be used against computer contagion can be learned from biology. Immune effectors of plants and animals protect against a broad range of pathogens; however, in nature this system evolved over millions of years. Engineering protective computer systems with similar efficacy within a few years is a great challenge. Current protection programs mainly resemble innate immunity, but programs that learn from exposure (thus resembling adaptive immunity) are under development. Vaccination with relatively harmless microbes primes the immune system. Biological hosts also naturally carry protective microflora that compete with pathogens. Could we produce “virtual vaccines” that are beneficial to the computers carrying them (e.g., by blocking preferred sites of entrance for viruses or repairing viral damage automatically) and let these “good” microbes circulate on the Internet just as malignant viruses do? Crude versions of such vaccines have already been developed. Recently, a worm by the name Fixing the Holes was discovered that utilized known security holes to spread to other hosts. Using “good” microbes would have its costs: occupation of Internet capacity and consequent slowdown of data transmission and presence of malicious worms disguised as beneficial ones to elude detection. 

 Knowledge of infectious diseases may help control computer contagion. Conversely, study of computer malware may help curb infectious disease emergence. Internet contagion illustrates how pathogens emerge and spread in our increasingly small world. The speed of virtual pathogen evolution makes it possible to follow the process of mutation and selection in real-time. With countless interlinked computers, the risk for virtual contagion is so great that urgent steps are needed to avoid catastrophe. How many pandemics will it take before we accept the risks and costs of computer immunity? Similarly, to protect against emerging pathogens, we must use all tools available, including virtual pandemics. A task force to collect data on the epidemiology of virtual infections as a model for infectious diseases might be an important first step.

